# A Social Network Analysis Approach to COVID-19 Community Detection Techniques

**DOI:** 10.3390/ijerph19073791

**Published:** 2022-03-23

**Authors:** Tanupriya Choudhury, Rohini Arunachalam, Abhirup Khanna, Elzbieta Jasinska, Vadim Bolshev, Vladimir Panchenko, Zbigniew Leonowicz

**Affiliations:** 1 Informatics Cluster, School of Computer Science, University of Petroleum and Energy Studies (UPES), Dehradun 248007, India; 2 Miracle Educational Society Group of Institutions, ViziaNagaram 535216, Andhra Pradesh, India; rohinaruna@gmail.com; 3 Systemics Cluster, School of Computer Science, University of Petroleum and Energy Studies (UPES), Dehradun 248007, India; abhirupkhanna@yahoo.com; 4 Department of Operations Research and Business Intelligence, Wrocław University of Science and Technology, 50-370 Wroclaw, Poland; elzbieta.jasinska@pwr.edu.pl; 5 Federal Scientific Agroengineering Center VIM, 109428 Moscow, Russia; pancheska@mail.ru; 6 Department of Theoretical and Applied Mechanics, Russian Open Academy of Transport, 125315 Moscow, Russia; 7 Faculty of Electrical Engineering, Wroclaw University of Science and Technology, 50-370 Wroclaw, Poland; zbigniew.leonowicz@pwr.edu.pl

**Keywords:** clustering, social network, COVID-19 community, node metrics

## Abstract

Machine learning techniques facilitate efficient analysis of complex networks, and can be used to discover communities. This study aimed use such approaches to raise awareness of the COVID-19. In this regard, social network analysis describes the clustering and classification processes for detecting communities. The background of this paper analyzed the geographical distribution of Tambaram, Chennai, and its public health care units. This study assessed the spatial distribution and presence of spatiotemporal clustering of public health care units in different geographical settings over four months in the Tambaram zone. To partition a homophily synthetic network of 100 nodes into clusters, an empirical evaluation of two search strategies was conducted for all IDs centrality of linkage is same. First, we analyzed the spatial information between the nodes for segmenting the sparse graph of the groups. Bipartite The structure of the sociograms 1–50 and 51–100 was taken into account while segmentation and divide them is based on the clustering coefficient values. The result of the cohesive block yielded 5.86 density values for cluster two, which received a percentage of 74.2. This research objective indicates that sub-communities have better access to influence, which might be leveraged to appropriately share information with the public could be used in the sharing of information accurately with the public.

## 1. Introduction

Coronaviruses are a large family of viruses that can cause illness in animals and humans. In humans, several coronaviruses are known to cause respiratory infections, ranging from the common cold to more severe diseases, such as Middle East respiratory syndrome (MERS) and severe acute respiratory syndrome (SARS). The most recently discovered coronavirus causes coronavirus disease COVID-19. In December 2019, a coronavirus outbreak occurred in China. The WHO identified the type of virus as SARS-CoV-2, which affects the respiratory tract, such as the nose, throat, lungs, and windpipe, and can result in a severe attack of pneumonia and death. The most common symptoms of COVID-19 are fever, fatigue, and a dry cough. Some patients may have aches and pain, nasal congestion, runny nose, sore throat, or diarrhea. These symptoms are usually mild, and begin gradually. Some people become infected, but do not develop any symptoms nor feel unwell. Throughout the pandemic situation of COVID-19, the number of confirmed cases has increased around the world; approaches for COVID-19 solutions have received a great deal of attention from big pharma companies and doctors, and researchers have to deal with increasing volumes of day-to-day cases. It has been a challenge to identify means for processing, preparing, and responding against the virus.

Data science has recently attracted attention for solving issues in various domains, such as medicine and engineering. In the medical field, AI plays an important role in the diagnosis and prediction of the accuracy of large samples. However, few studies have focused on the propagation of awareness that helps health care people make decisions regarding the outbreak of the disease. Behind the main force of AI to analyze big data is machine learning, which detects the frequency of patterns and insights, and predicts outcomes. AI algorithms can then be used to determine the risk of cross-infection and alert users to such risks. The Facebook social network platform has created millions of actors that interact and group with each other. Their links are based on relationships between friends, colleagues, or are based on their mutual interests, shared tags, etc. In the context of COVID-19, community link computations of dynamic social networks have the unique property of recognizing particular information being exchanged in the content in the context of COVID-19. Researchers or social network analysts analyse data to determine the bad and good effects of the review outline of accurate information on COVID-19 infection, individual safety, and public health. Social media serves as a potential tool for people to change their panic behavior. A challenging problem in this domain is the promotion of welfare through the community. The discovery of a community of links is based on modularity. The objective is to find the target users of cohesive relationships in bipartite networks so that word-of-mouth and information transmission may be manipulated among friends, friends-of-friends, and followers-of-friends. Finally, community detection is a key challenge in the computation of propinquity links, and is related to the clustering of COVID-19 communities. In the proposed study, we are concerned with the analysis of interactions among communities on social media, and the mesoscopic features of the structural patterns of links. It is a widely used platform to provide awareness to users, and a fast-growing means of getting information for end users. This study aims to show the means by which social network data analysis discovers patterns of node participation and relates user participation patterns to their behaviors. The objective is to find the large users of cohesive relationships in bipartite networks so that word-of-mouth and information transmission may be manipulated among friends, friends-of-friends, and followers-of-friends in structural networks. The contributions of this research are as follows: Section 2 describes the background study of related works; Section 3 defines the search techniques used to analyze the mesoscopic features of public health care communities on Facebook; and in Section 4, we analyze the node centrality metrics that have been developed to determine the relative importance or centrality of a node. The proposed research clusters the links and predicts the trusted links based on the security conditions. It presents the steps to perform the iteration values of the nodes and determines the distance and centroids of clusters in the homophily structural network of the COVID-19 public health care parameter data set. The conclusions of this study are presented in Section 5.

## 2. Background of the Study

Exploratory data analyses of the epidemiological breakout of coronavirus studies have focused on the visual data analysis of its time-series function [1]. The collected spatial data sets of COVID-19 cases in China were divided into city and country-level pandemic areas. The static data on new infections, as well as daily recoveries, mortality cases, and the cumulative spread of infections, were evaluated of new infections was analyzed The machine-readable data set analyzed and summarized the estimates of population, ethnicity, employment, income, education, and other related metrics [2]. Significance analysis of emotions was performed to understand the issues of COVID-19 in order to make decisions. The analysis fell under the categories of text mining, graph data mining, and machine learning of mathematical models [3,4]. Text mining can be used to discover coronavirus trends in social media data. Users with social networks share information and ideas. It has been revealed that node concerns differ by discipline, and that their interactions between communities can be observed [5]. The time complexity of the interactions between users was analyzed using a network embedding algorithm [6]. The matrix factorization procedure, which uses weighted directed and undirected linkages, was utilised to minimise the lengths of pathways utilising the neighbourhood nodes of a unified framework and speed-up strategies links [7]. The intrinsic links between the dyadic pairs of the scalable method were obtained by the strength of cohesion between the nodes in the structure. The weighted links were compared with social network models [8]. The nodes by centrality metrics were measured using the weight-based approach between the strong ties in the structural network [9,10]. In The interactions of messages were grouped into frequencies of messages that were ordinary activities, customization of messages, and the regularity of messages they experimented with in order to assess the accuracy of linkages; this strategy gave greater accuracy than previous models [11]. The framework of influence propagation and diffusion between the strong links of homophily networks was analyzed based on the structural properties of the mesoscopic features of data mining [12]. The influential target node focuses more on the content of information; uniqueness, quantity, and innovativeness [13] were considered the centrality of the network and the characteristics of information diffusion [14]. We examined the factors influencing online social networks [15,16,17,18,19,20]. According on similarity of features in clustering approaches, diffusion was thought to be a result of the heterogeneity of social networks and has been linked to information spread. Regression analyses were used to analyze the similarities of nodes [21,22,23,24]. The undirected graph of the node interacts among (n − 1) nodes of the similarity of the degree of the links. The analyzed values of each community yielded better accuracy [25,26,27,28,29]. Machine learning in graph theories solved social network issues and deals with the applications of machine learning techniques [30].

## 3. Materials and Methods

**Figure 1:**
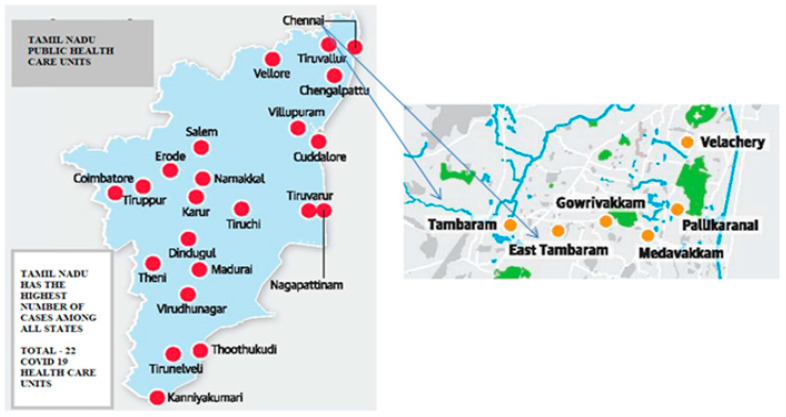
Distributed map of public health care centers in Chennai city, Tambaram zone.

**Figure 2:**
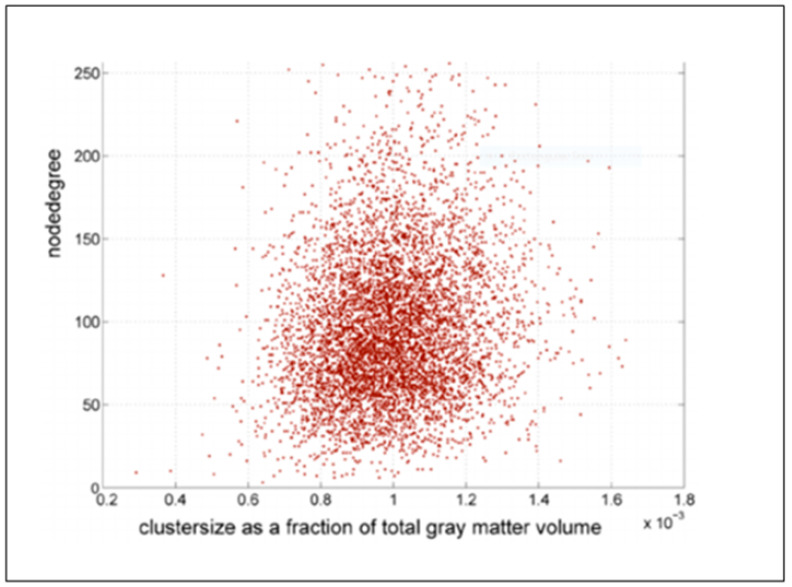
Cluster nodes.

**Figure 3:**
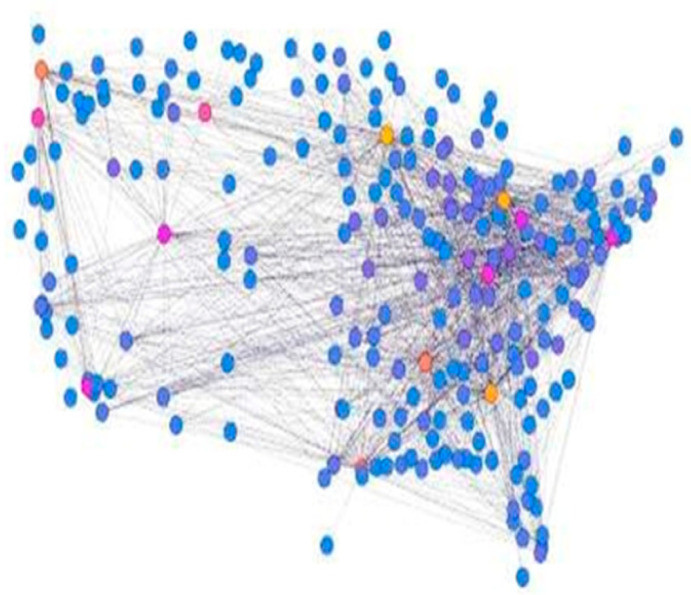
The layout of the 100 nodes.

**Figure 4:**
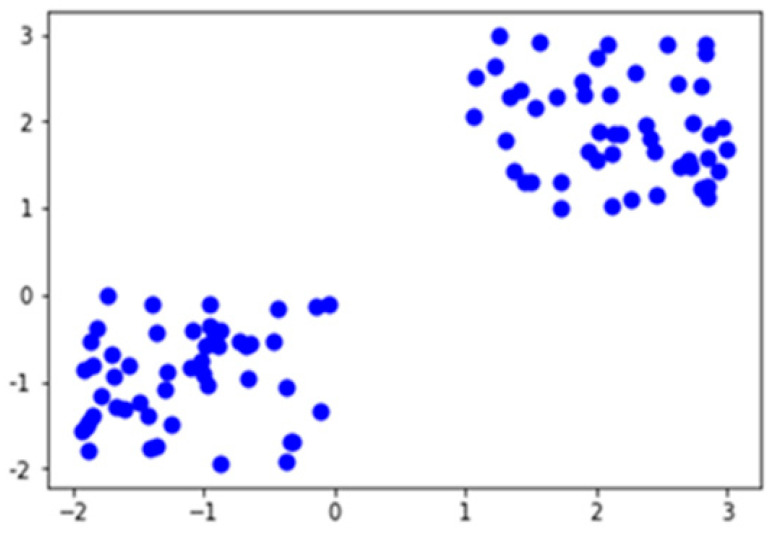
Segmentation of the cluster.

**Figure 5:**
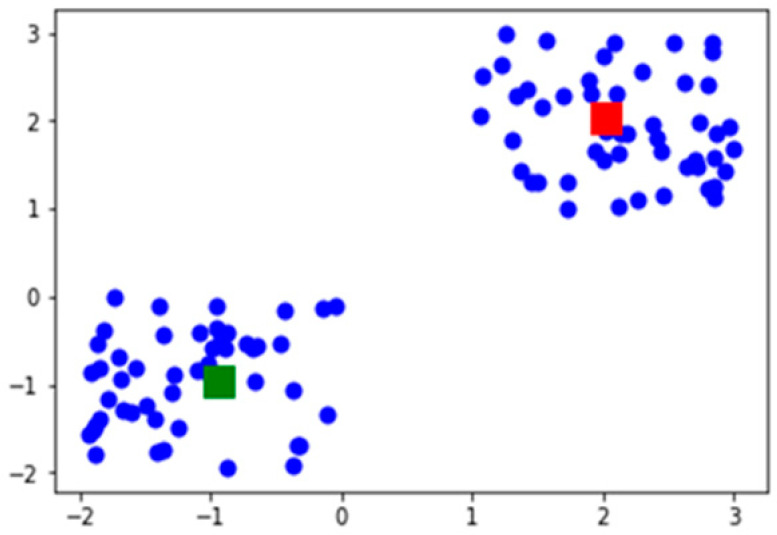
Centroids of the cluster.

The study period was four months in the Tambaram zone in 2020, which was analyzed by the number of COVID-19 notified cases. A map of the study area is shown in Figure 1. It is a thematic map of nodes that pays close attention to nodes shown in Figure 2, Figure 3, Figure 4 and Figure 5 shows the thematic map of nodes.

### 3.1. Approaches for Searching Strategies

Two statistical search techniques are adopted to analyze the mesoscopic features of public health care communities on Facebook, In the following section we analyze the shared communication data, which was performed using the breadth-first search and uniform search techniques. We did not review and analyze the COVID-19 word-of-mouth and interactive information about the nodes, nor the connections between friends-of-friends and followers-of-friends lists that are publicly accessible. For this purpose, learning knowledge analytics was designed with user pages, and connections were extracted.

#### 3.1.1. Breadth-First Search Technique (BFS)

A graph is a collection of vertices that are connected by links. Vertices in the graph explore the neighborhood seeds of each node; it visits the follower of neighbors and so on until the entire structure has been visited. This technique has the advantage of the random walk method. According to the heuristically based calculation of the communities of nodes in the growth rate during the sampling of search techniques, the results show the determination criterion of the mining process of 15 days of running time, by observing that the network structure has less than 20% growth overall. The obtained size of the homophily graph was used as a yardstick for the uniform search technique.

To retrieve the nodes visited, the recursive function was applied to a linked undirected graph node designated “By then all vertices in B,” and the graph traversal was performed until the node connected with the end-of-node in the homophily network. The complete traversal of the graph can be achieved by repeatedly calling the BFS each time with a new unvisited vertex. Algorithm 1 is called by followers-of-followees in the structural network. Two statistical search techniques were adopted to analyze the mesoscopic features of COVID-19 public health care communities.
**Algorithm 1**: Explored the nodes by breadth-first techniqueInput: seed S, node “i”,Visited [i] = 1 already the node to be visited.Visited [i] has initiated to Zero.Output: Get Unexplored Seed{U = S//Q Vertices in the QueueVisited [S] = 1;Repeat{Λ vertices w acting from U doIf (visited [w] = 0) then{Add w to q;//w is unexploredVisited [w] = 1;}}If q is empty, then return unexplored vertex;Remove S from Q; }//until Q is not explored.

#### 3.1.2. Uniform Search Technique (US)

The details of Facebook users was analyzed by the US technique. The structural properties generated a node ID distributed randomly in the user’s system, and showed that the maximum number of assigned Node IDs was 1000 in the space of 32-bit numbers as of January 2020 (the period in which we carried out the sampling technique; the number of subscribed users on FB was 5000). The probability of randomly generating an existing node ID is ~1/6. The search technique was set as follows: the generated number of node IDs in the range of 216 is equal to the dimensions of the network size. Our expectation was to obtain comparable sample dimensions using the breadth-first search technique.

### 3.2. Metrics for Centrality

One of the key applications of social network analysis (SNA) is to distinguish the most vital or focal nodes in the system. Various centrality metrics have been developed to determine the relative importance or centrality of a node. The most commonly used metrics are degree centrality, betweenness centrality, eigen centrality, density, clustering, and closeness centrality.

#### 3.2.1. Degree Centrality Metric

Degree centrality compares the in-degree and out-degree of links; the node with the highest degree is the most highly ranked. The nodes have direct connections and the edges in the graph are distinguished by the in-degree of the linkages, this may be used to determine the nodes that most likely directly affect the greatest number of other nodes. The number of edges is directed towards the centrality of the node. In order to find the connected edges of frequently visited nodes, degree centrality indicates the number of connections that identify the most and least active nodes. Friends are likely to save the information content of these nodes in order to connect with other nodes. The simplest metric of in-bound and out-bound links for active propagating information link transactions.

#### 3.2.2. Eigen Centrality Metric

This metric measures the closeness and influence of the source and target nodes in a network. The active node receives a degree whenever the node points increase as a result of the sharing of information. However, not all nodes in a network can be considered equivalent; some nodes are relevant, and others may or may not be. Thus, an active node should have more influential links than highly linked links; the calculated values of the eigenvector identify the closeness values of the egocentric nodes.

#### 3.2.3. Closeness Centrality Metric

The centrality of the closeness metric scores each node based on the proximities of all other nodes in the network. It assigns a score to each node to determine the shortest path between nodes in the community. A central node helps to propagate information in the connected network, and it may be more useful to find influencers within a single cluster.

#### 3.2.4. Density

The density of the network is defined by the number of connected links divided by the total number of edges that could exist in the structure; this metric reflects the strength of the strong links in a group of nodes and indicates the graph structure.

#### 3.2.5. Clustering

Clustering social ties is challenging, and the proposed study talks about finding the centroid of the community for information propagation, and analyzing aggregation patterns and social dynamics to detect and examine online communities. The acquisition of data sets from Facebook COVID-19 public health care synthetic networks was undertaken to analyze the clustering. This study discusses in detail various mesoscopic features of the community structure of COVID-19 public health care networks. In order to analyze the links, software application R Programming 4.0.3 was used. This application has a large number of packages for statistical analysis using the I-graph package; other features were visualized using the ggplot2 package.

Facebook COVID-19 public health care synthetic networks contain hundreds of users and their social relationships, after which the study discovered communities that represent the clusters or aggregated units among network members. The study analyzed the community’s statistical properties and found and characterized some specific ideas which were followed by health care members. The term clustering indicates the arrangement of smaller and stronger groups with numerous edges. For example, in an interpersonal organization, there are individuals frame clusters in which every part is known as an alternate individual; grouping in this way frames one of the essential qualities of certifiable systems and, accordingly, numerous measurements for estimating it can be characterized. The local clustering coefficient of a node is defined as the probability that two randomly chosen (but distinct) neighbors are connected in the groups.

#### 3.2.6. Periphery Structure of the Upper and Lower Bounds

The upper and lower bounds on the γ-clique number in the periphery structure are generalizations of the nodes. The γ-clique number ω of a graph G with m vertices and n edges satisfies the following inequality in Equation (1):

(1) $$\omega \left(G\right)\leq \gamma +\sqrt{\gamma^{2}+8\gamma m/2\gamma }$$

The first bound Equation (2) is obtained by solving the quadratic inequality:

(2) γ(ω(G)(ω(G−1)/2≤m

Assuming that graph G is connected and has a γ-clique size, the following inequality must hold:

(3) γ(ω(G)(ω(G)−1)/2)+n−ω(G)≤m

After solving this quadratic inequality for ω (G), Equation (2) obtains the upper bound values for the node, while the lower bounds of the node have walked randomly in the peripheral structure of the network for Equation (3).

#### 3.2.7. Relationship

If the inequality rises, the relationship of the in-degree and out-degree of the links that will relate to the γ-clique number to the node has randomly walked in the group, as well as in the clusters, to create the relationship between the two clusters. These strong links act to influence the communities in the synthesized structural network. The classical bound techniques for the node relationship *ω* (*G*) are easily obtained from the following formulation for the maximum random walks in the dynamic interaction:

(4) $$\omega \left(G\right)=\frac{1}{1-\rho }$$

where ‘ρ’ is the number of nodes in the group.

#### 3.2.8. Simulated Data

By using uniform search technique, Table 1 was analyzed. A total of 1000 nodes were discovered with their total edges being 2000 by measuring the completeness diameter of the structure links as 5.69, and the total number of possible edges between all the node values as 0.435%. The normalized cut of the connected components in a uniform search was 600 edges, from 1000 nodes. The average internal degree of all the node values was 296.8. The similarity and the influence based on the correlation of the closeness value was 48.93% for 600 edges. Consequently, the breadth-first search technique of 1200 nodes with connected component edges in the structure was 680, and their closeness metric of the average degree of the link was 278.5, owing to the lower density value of 0.358; hence, the clustering coefficient value is higher than the uniform search value of 0.369. The very influential linkages in the community were given to the active nodes with an eigenvalue of 25.46 percent, which was lower than the uniform search strategy. The United States is heavily connected to the community’s egocentric nodes, and the COVID-19 public health care awareness linkages spread quickly to other users. The dissemination of information occurred in the United States; however, these links may not be trusted because they are unable to easily identify target users in the connected components of 680 homophily network nodes, which are required for the cognitive closure of visited nodes among 655 nodes in the community. It received the highly influential links in the community of the eigenvalue community.

The clustering coefficient of BFS is 0.369, with a diameter of 5.86; the nodes reciprocate with each other in the interaction of a linear combination of time and emotional intensity. By comparing the metrics of BFS and US among the total number of users (1000), the search technique was applied. The US produced better results in terms of density and clustering coefficient than the BFS.

## 4. Results & Discussion

Clustering depends on the similarity of nodes for a particular purpose. For ‘n’ number of nodes, the segmentation of the method constructs ‘k’ partitions of the nodes, where each node represents a cluster k < n, which is the node satisfied and classified into “k” groups. The following requirements satisfy the fuzzy partition techniques: each sub-community in the cluster must contain at least one node; the sub-community of any one of the nodes belongs to the existence of any one of the groups in the cluster; the number of partitions ‘k’ has initiated to the start seed of the cut-ratio. Iteratively, the node has attempted to expand the partition by the single node, but overlaps from one group to another. Generally, the cohesion of a node in a similar group is linked to each other in the cluster, as this part of the criterion judges the quality of the links in the cut ratio. Our proposed technique has an optimal portioning-based cluster technique that enumerates all possible partitions, and a popular heuristic method has been adapted instead of other possible techniques.

### 4.1. Partition Algorithm

It represents the mean values of the nodes in the cluster and is located near the centroid by one of the nodes.

The parameters “k” of COVID-19 public health care nodes, and their partition sets of the tendencies of each attribute were analyzed to associate and link with similarities of “n” nodes into “k” clusters so that the resulting intra-cluster similarity was higher than the inter-cluster similarity. Partitioning was based on the mean values of the parameters of the health care nodes.

(5) E=∑(k−1)n=[∑∣m−Ci∣^2]

“E” is the squared error of the nodes, “k” is the point in the structure of the node, C_i_ is the cluster, and “m” is the mean of the cluster. At random, 100 nodes were taken into the analysis and divided into two clusters named as “Cluster One” and “Cluster Two”. Each cluster of a node had strong cohesive bonds in each partition.

### 4.2. Initial Cluster

Table 2 provides more information about the random centroid of the clusters. From among all the nodes that had unique IDs, the weighted values of all the nodes were tabulated to identify the weak and strong ties of the links. The centrality of the links was common for all IDs. First, we analyzed the distance between the nodes for segmenting the sparse graph of the groups. Bipartite structure was taken into the segmentation of the sociograms of 1–50 and 51–100 and was divided based on the clustering coefficient values. For each of the nodes, the nearest cluster was identified, and tabulated values are shown in Table 2. The (x) values of 14–39 were grouped into the nodes in Cluster One, and the rest of the nodes were grouped into the second cluster based on the random centroid values. With Equations (3)–(5), the peripheral structures of the lower and upper bounds were calculated in order to identify the relationship with the node. After the node was grouped, the results yielded for the centroid a value of 23.68% for Cluster One, while the second yielded 72.91%. By the associated values not visited and the follower-of-followee links not visited, the neighborhood node by the structural diameter values of US was 5.69. The first cluster of nodes did not reach the bottom of the heuristic search strategy. Randomly, the node clustered but it was not a strong cohesion between ‘n’ nodes, and it identified the weak ties of the links; if the information propagated to the users, it would not reach all the nodes. Some structural holes took place in Cluster One. In order to fill a structural hole, the node was iteratively analyzed at 15.33 degrees; the results are tabulated in Table 3. The two clusters of the individual score included two variables: the initial partition is the initial means of the cluster for each node; in the outlier, if any individual nodes are located, the individual node of the active links is added. In summation, the values are individual. Individual nodes (X) in cluster (C) were divided into the sum of the cluster score values, and the second cluster was determined as follows: the average comparison was taken from the second cluster, and the scores were summed; if the average value was closer to the mean values of Cluster One, we performed the steps for the second cluster, compared each node distance to its origin of the cluster’s mean vector and to that of another cluster; the mean vector should be similar to its distance (D1) to the other vertex; if the node is not, relocated Algorithm 2 continues the steps if no relocation occurs to stop the iterative process, and clustering is completed.
**Algorithm 2:** K-means partitionInput: clusters “k” and it contains “n” number of nodes.Output: A set of cluster “k” minimized the squared error.Method1. Randomly choose the objects (k) in the initial cut.2. Repeat3. Similar mean values of the node have been assigned to the cluster.4. Update the cluster after assigning the new node.5. until the changes do not occur in the group.

Table 3 depicts the iteration values of the nodes to find the distances and centroids of the cluster in the homophily structural network of the COVID-19 public health care parameter data set. All the nodes have unique IDs; the experimental results of two clusters and their two centroid values are 32.68% and 74.2%, compared to iteration 1 and iteration 2, respectively. Iteration 2 yielded good performance for modularity; the edges were communicated with high scores of centroid values, 32.68% in Cluster One and 74.2% in Cluster Two. The internal edges were more than the expected random walk of all nodes based on heuristic strategies. This preserves the degrees of distribution in the network. Degree centrality displays the amount of connections that identify the most and least active nodes in order to determine the associated edges of often visited nodes. In order to connect with other nodes, friends are likely to preserve the information content of these nodes in the iteration 2.

### 4.3. Structural Analysis

The structural analysis involved fetching actual Facebook data samples that were statistically analyzed using the R programming tool, obtaining the performance results of the clustering of nodes, and determining the centroid values of the cluster. The spatial locations of nodes were required for the analysis of neighborhood relationships. Network metrics quantitatively measured the mesoscopic structural properties of the network as well for the identified communities, whereas the performance metrics of network analysis using R software programming quantitatively compared properties of these communities against the ground-truth communities. Demographic properties were analyzed and clustered to identify the communities in the structural network. Social networks also create communities between the nodes of synthetic social networks. Thus, spatial information of the two clusters was identified based on the similar behaviors of the nodes, and their internal edges were strongly connected in the community.

Figure 3 shows the layout of 100 nodes. An undirected graph is decomposed into bounded nodes. This reduces computational cost and renders it scalable. This study’s applications are particularly useful for facilitating fast communication in large locations using influential nodes. The colors yellow and purple represent the influential target nodes of the cluster; the information is propagated by target users and holds a high degree of value compared to all other nodes in the network.

Figure 4 shows segmentation based on the cut-ratio we segmented the two clusters into for the structural network. The average internal density was even across all the nodes; the possible numbers of edges were clearly in the two clusters had to communicate between the nodes.

Figure 5 depicts the two centroids between the two clusters, shown in green and red node points. The fraction over the median degree was greater than over the internal degree of the nodes. It is the normalized cut-off edges of the equal partitioning of the nodes. The links are analyzed by the 0 and 1 values of the active and inactive links in the cluster, for 100 nodes of the array values that are given below. The data points are categorized into the following:

Array [0, 0, 0, 0, 0, 0, 0, 0, 0, 0, 0, 0, 0, 0, 0, 0, 0, 0, 0, 0, 0, 0, 0, 0, 0, 0, 1, 1, 1, 1, 1, 1, 1, 1, 1, 1, 1, 1, 1, 1, 1, 1, 1, 1, 1, 1, 1, 1, 1, 1, 1, 0, 1, 1, 1, 0, 0, 0, 0, 0, 0, 0, 0, 0, 0, 0, 0, 0, 0, 1, 1, 1, 1, 1, 1, 1, 1, 1, 1, 1, 1, 1, 1, 1, 1, 1, 1, 1,1, 1, 0, 0, 0, 0, 0, 0, 0, 0, 0, 0, 1, 1, 1, 1, 1, 1, 1, 1].

The community framework is an innovative research area for COVID-19 public health and medical care. It could be possible for researchers to improve the nodes to decide the self-awareness of the public individual by exploiting these online social media networks or many social data sets in order to find patterns or ideas in the spread of information within this network itself. On the same ground, exploiting users’ affiliations towards their connected communities could be effective to predict useful data patterns to recommend COVID-19 public health care guidance and awareness, based on this notion of common similarities shared within the communities. In order to find communities, a particular user affiliation can be used as an important characteristic; this can be achieved by maximizing the resolution limit to 1 as a constraint. Given the optimization of modularity with few limitations, for example, based on the layout of the network, the study could not find a community whose size is smaller.

**Figure 6:**
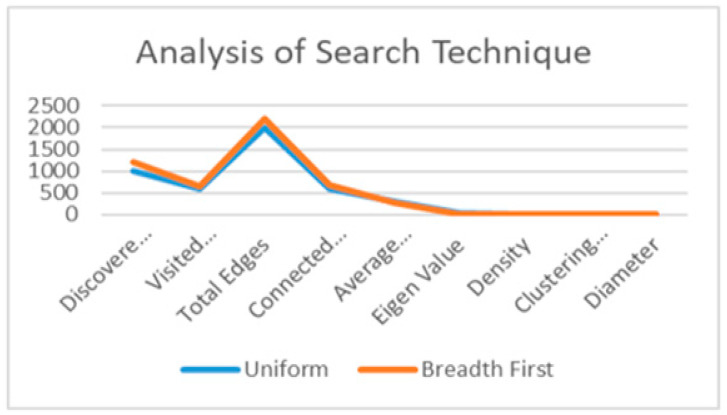
Comparison of uniform search and breadth-first search techniques.

A hierarchical-based circular algorithm was used to represent the community framework. From this representation, the study could see the strong links between communities, which occupy a center core position in the graph network; their positioning does not depend on size. This shows that many small communities, rather than big communities, play a crucial role in the network; moreover, they exist in the center of the network and hold for the network periphery. The study discovered that the number of edges for each community varied depending on its size, indicating that small communities are more linked between clusters and have less interconnectivity both within and outside of the group. However, users of large or larger structural networks are less connected with each other despite strong components or links that exist in large communities as a consequence of their greater numbers. In Figure 6, a comparison of the BFS and US search techniques shows that the US strongly found the community by the parameters of the outcome, with an average of 489 for the US, and 481 for the BFS. Therefore, structural holes are high in the BFS. The eigenvalue of BFS was 89, while that of US was 84, and the clustering coefficients of both BFS and US were 56. The densities of the clusters were the same for BFS and US, with a 3-diameter distance of 2000 nodes. While the BFS discovered the edges to analyze 1500 from the 2000 total edges, spatial information was spread less in the community. The US has stronger nodes and spread the information to 1020 nodes out of the 2000 total edges.

Accordingly, the node was used for analyzing the uniform search values for all analyses of cluster partitioning methods of dynamically chosen nodes and random walks of nodes of the upper and lower periphery of the synthesized structural network. The US technique effectively provides the results of finding the centroid of the nearest cluster values.

## 5. Conclusions

The study demonstrated social network analysis (SNA) using COVID-19 public health care links of the Facebook awareness community. This approach employed the task of grouping a node by some measures of link similarity, degrees of links, and criteria for cluster partitioning on complex networks. As a result, the study found the spatial information of the community to be much more suitable in terms of COVID-19 public health care links. It is expected that the current study could be an important reference for exploring the content and analyzing influential nodes in order to expediently propagate the awareness of disease, and raise prevention and health promotion strategies; SNA is a more logical and objective approach than others to do so. The K-means algorithm was used to partition the data into two clusters based on the weighted edges of a node in the dynamic walk of the upper and lower peripheries of the structure. Cluster Two received 74.2% of higher access to influence propagation in the sub-communities associated with the structure. This study analyzed the spatial information of a methodological strategy for social network analysis, specifically in the case of social rights protection in COVID-19 public health care. The research could help facilitate the sharing of information with very similar demographic characteristics of nodes, which could be related to the analysis of proper awareness and informing of the public.

## Figures and Tables

**Table 1:** Comparing network measuring metrics of the community.

Feature	Uniform Search	Breadth First Search
**Discovered Nodes (N)**	**1000**	**1200**
**Visited Users (V)**	**598**	**655**
**Total Edges (E)**	**2000**	**2200**
**Connected Components (C)**	**600**	**680**
**Average Degree (A)**	**296.8**	**278.5**
**Eigen Value (E)**	**48.93**	**25.46**
**Density (μ)**	**0.435%**	**0.358%**
**Clustering Coefficient (C)**	**0.263**	**0.369**
**Diameter (D)**	**5.69**	**5.86**

**Table 2:** Iteration 1—find the centroid values of the nearest cluster.

X	C	D1	Nearest Cluster	Centroid
14	13	1	1	23.68
15	13	2	1	
16	13	3	1	
15	13	2	1	
12	13	−1	1	
14	13	1	1	
20	13	7	1	
21	13	8	1	
25	13	12	1	
24	13	11	1	
23	13	10	1	
29	13	16	1	
40	13	27	1	
35	13	22	1	
37	13	24	1	
39	13	26	1	
54	13	41	2	
56	13	43	2	
53	13	40	2	72.91
60	13	47	2	
64	13	51	2	
68	13	55	2	
75	13	62	2	

**Table 3:** Iteration 2—find the next closest centroid values of the nearest cluster.

X	C	D1	Nearest Cluster	Centroid
16	15.33	−1.33	1	
17	15.33	−0.33	1	
18	15.33	0.67	1	
17	15.33	−0.33	1	
14	15.33	−3.33	1	
16	15.33	−1.33	1	
22	15.33	4.67	1	
23	15.33	5.67	1	
27	15.33	9.67	1	
26	15.33	8.67	1	
25	15.33	7.67	1	
31	15.33	13.67	1	32.68
42	15.33	24.67	1	
37	15.33	19.67	1	
39	15.33	21.67	1	
41	15.33	23.67	1	
56	15.33	38.67	2	
58	15.33	40.67	2	
55	15.33	37.67	2	
62	15.33	44.67	2	
66	15.33	48.67	2	
70	15.33	52.67	2	74.2
77	15.33	59.67	2	
81	15.33	63.67	2	
84	15.33	68.67	2	
88	15.33	72.67	2	
94	15.33	78.67	2	
100	15.33	84.67	2	

## Data Availability

Data generated at a central, large-scale facility, available on the open source of Primary Healthcare Details during COVID-19.
